# Bioactive Properties of Nanofibres Based on Concentrated Collagen Hydrolysate Loaded with Thyme and Oregano Essential Oils

**DOI:** 10.3390/ma13071618

**Published:** 2020-04-01

**Authors:** Mariana Daniela Berechet, Carmen Gaidau, Aleksandra Miletic, Branka Pilic, Maria Râpă, Maria Stanca, Lia-Mara Ditu, Rodica Constantinescu, Andrada Lazea-Stoyanova

**Affiliations:** 1 Division Leather and Footwear Research Institute, National Research and Development Institute for Textiles and Leather, 031215 Bucharest, Romania; marianadanielaberechet@yahoo.co.uk (M.D.B.); maria.alexandu@gmail.com (M.S.); rodica.roxana@yahoo.com (R.C.); 2 Faculty of Technology, University of Novi Sad, 21102 Novi Sad, Serbia; alexm@uns.ac.rs (A.M.); brapi@uns.ac.rs (B.P.); 3 Center for Research and Eco-Metallurgical Expertise (ECOMET UPB), Politehnica University of Bucharest, 313 Spl. Independentei, 060042 Bucharest, Romania; 4 Faculty of Biology, University of Bucharest, 60101 Bucharest, Romania; lia_mara_d@yahoo.com; 5 National Institute for Lasers, Plasma and Radiation Physics, 077125 Magurele, Romania; andrada@infim.ro

**Keywords:** antimicrobial nanofibres, collagen hydrolysate, essential oils, bioactive nanofibres, biofilm eradication, alternative antimicrobials, electrospinning technique

## Abstract

This research aimed to obtain biocompatible and antimicrobial nanofibres based on concentrated collagen hydrolysate loaded with thyme or oregano essential oils as a natural alternative to synthesis products. The essential oils were successfully incorporated using electrospinning process into collagen resulting nanofibres with diameter from 471 nm to 580 nm and porous structure. The presence of essential oils in collagen nanofibre mats was confirmed by Attenuated Total Reflectance -Fourier Transform Infrared Spectroscopy (ATR-FTIR), Ultraviolet–visible spectroscopy (UV–VIS) and antimicrobial activity. Scanning Electron Microscopy with Energy Dispersive Spectroscopy analyses allowed evaluating the morphology and constituent elements of the nanofibre networks. Microbiological tests performed against *Staphylococcus aureus, Escherichia coli, Pseudomonas aeruginosa* and *Candida albicans* showed that the presence of essential oils supplemented the new collagen nanofibres with antimicrobial properties. The biocompatibility of collagen and collagen with essential oils was assessed by in vitro cultivation with NCTC clone 929 of fibroblastic cells and cell viability measurement. The results showed that the collagen and thyme or oregano oil composites have no cytotoxicity up to concentrations of 1000 μg·mL^−1^ and 500 μg mL^−1^, respectively. Optimization of electrospinning parameters has led to the obtaining of new collagen electrospun nanofibre mats loaded with essential oils with potential use for wound dressings, tissue engineering or protective clothing.

## 1. Introduction

In recent years, special attention has been paid to the electrospinning process as a simple method of making nanofibre-based structures. Due to the high porosity and area/volume ratios of nanofibres, different active substances with specific properties and with applications in food packaging [1], medicine [2], pharmaceutical drugs [3], fibres based electronics [4] or water treatment [5] can be included.

Electrospinning is a versatile technique for obtaining nano- or micrometric fibre-based mats with variable properties by applying high voltages between the tip of the injector needle (+) and a support fixed to a metal collector (−) on which they are deposited. In general, the electrospinning process involves a polymer that generates the carrier matrix consisting of continuous nanofibres, in which the presence of a network of high molecular weight facilitates the formation of a stable jet during electrospinning. However, in the last decade research has been made on electrospinning of polymer-free materials with functional molecules for other new applications, such as phospholipids [6], ammonium gemini surfactant [7], cyclodextrin derivatives [8] and tannic acid [9].

Nanofibre membranes made by electrospinning take precedence over other materials due to their high porosity and interconnectivity [10]. This structure permits the introduction of active substances giving the possibility for further functionalization [11]. 

Collagen is a biopolymer with regenerative and tissue reconstruction properties used in various treatments in medicine [12], drugs [13] or urinary drains [14]. In addition, collagen is used as food supplements [15] and antimicrobial emulsion formulations for cosmetic purposes [16]. 

Recently, the use of collagen loaded with bioactive compounds derived from plants for design of natural wound dressings classified as sponges, electrospun nanofibre matrices, films and hydrogels was investigated to overcome the toxicity and reduced antimicrobial activity of existing products [17]. In the present paper, collagen hydrolysate was used in high concentration due to the associative properties of collagen hydroysate molecules with effect on increased viscosity and improved electrospinnable properties. As compared to gelatin, collagen hydrolysate represents a more versatile, cheaper and available biomaterial. The combination of concentrated collagen hydrolysate with essential oils for new wound dressing manufacture is an original approach for skin wound healing by cell proliferation stimulation and antimicrobial protection. The use of essential oils represents a natural alternative for synthesis antimicrobials with pathogen resistance potential for new non active wound dressings design.

Plant essential oils are gaining a wide interest in medicine, agriculture and food industry due to their antibacterial, antifungal, antioxidant and anti-inflammatory properties [18]. The beneficial effect of essential oils to promote wound healing was extensively described [19]. The antimicrobial activity of plant essential oils is attributed to their chemical structure, in particular the presence of hydrophilic functional groups, such as hydroxyl groups of phenolic components and/or lipophilicity of some essential oil components [20]. To overcome the essential oils volatility, different formulation techniques and methods have been developed, such as casting [21], emulsion ionic gelation [22] or encapsulation by electrospinning [23]. 

Although many studies have demonstrated the antimicrobial effect of essential oils and their bioactive compounds against a broad spectrum of pathogenic bacteria in food [24] and pharmaceutical applications [20], there are no publications that discuss their incorporation into high concentrated collagen hydrolysate by electrospinning.

Peiwen and Mele [25] prepared electrospun mats based on clary sage and black pepper essential oil combined with polylactic acid (PLA) dissolved in acetone solvent showing antimicrobial properties for biomedical applications as dressings. 

In another paper, cinnamaldehyde essential oil was incorporated inside chitosan/polyethylene oxide (PEO) nanofibre mats, without the use of a surfactant, to obtain new flexible scaffolds with antimicrobial properties against nosocomial infections [26]. The encapsulation of oregano alcoholic extract in Eudragit E100 (cationic copolymer based on dimethylaminoethyl methacrylate, buthylacrylate and methyl methacrylate) was performed and showed that the active substance delivery from electrospun mats was highly influenced by the polymer concentration [27].

The novelty of this paper consists in the successful preparation of collagen hydrolysate-based nanofibres loaded with essential oils, with potential use for natural wound dressings. The investigations on antioxidant and antimicrobial activities and the biocompatibility of electrospun collagen loaded with thyme or oregano essential oils showed the potential of new biomaterials as friendly alternative to similar materials based on more expensive native collagen, synthesis polymers, organic solvents and pathogen resistant antibiotics. 

## 2. Materials and Methods

### 2.1. Chemical Materials

The following chemical materials were used in this study: Thyme essential oil (*Thymus vulgaris*) from Steaua Divina-Santo Raphael SRL (Bucharest Romania), oregano essential oil (*Origanum vulgare*) from Solaris Plant SRL-Radix (Darmstadt, Germany), Alcalase 2.4 L (protease from Bacillus licheniformis with 2.4 U/g), 2,2-diphenyl-1-(2,4,6-trinitrophenyl)hydrazyl (DPPH), sodium carbonate (Na_2_CO_3_), gallic acid (C_6_H_2_(OH)_3_COOH) from Sigma-Aldrich, hydrogen peroxide (30% solution of H_2_O_2_, Mw = 34.01 g/mol) from Silal Trading SRL, (Bucharest, Romania), methanol anhydrous (CH_3_OH) from Chimreactiv SRL (Bucharest, Romania), calcium oxide hydrated (CaO CaOH, MW = 81.371 g/mol) from Cristal R Chim SRL (Bucharest, Romania) and pearl sodium hydroxide (NaOH, Mw = 40 g/mol) from Lachner (Neratovice, Czech Republic).

### 2.2. Preparation and Characterisation of Concentrated Collagen Hydrolysate 

The collagen hydrolysate was obtained by the alkaline-enzymatic hydrolysis of the decalcified bovine pelt in two steps [28]. In the first stage, the bovine pelt was hydrolyzed in 400% water (w/v) with 10% CaO (w/w), for 4 h at a temperature of 80 °C, under mechanical stirring. Then, the pH was adjusted to 8–9 value with 0.5% NaOH (v/v) solution of 20% concentration and 0.4% Alcalase 2.4 L (w/w) was added under stirring regime for 3 h, at 65 °C. After 3 h of enzymatic hydrolysis, the temperature was raised at 90 °C and it was maintained at this temperature for 15 min until the enzyme deactivation. Subsequently, the collagen hydrolysate was decanted, filtered and concentrated 1:4 in the Heidolph rotary evaporator (Schwabach, Germany) at a temperature of 65 °C, pressure of 150 mbar and 150 rot/min.

The physical–chemical characteristics were analysed according to standardized and in house methods: SR EN ISO 4684:2006 (dry matter), SR EN ISO 4047:2008 (ash content), SR ISO 5397:1996 (total nitrogen and protein), STAS 8619/3:1990 (pH), ICPI Method (aminic nitrogen) and SR EN ISO 27883:1997 (electrical conductivity). The viscosity of concentrated collagen hydrolysate was 1623 cP, measured with the Brookfield AMETEK DV2T TC-550 Viscometer at 25 °C. The size and Zeta potential of collagen particles were measured by Dynamic light scattering (DLS) technique with Zetasizer Nano-ZS device from Malvern (Malvern Hills, UK).

The results of the analyses are expressed as the average values of three determinations.

### 2.3. Essential Oils (EOs)

Thyme and oregano essential oils have been chosen because they are rich in bactericidal and anti-fungal monoterpene derivatives such as carvacrol and thymol (low molecular weight volatile components [19]). Due to these properties, the essential oils are used in preparations against skin infections, in the treatment of fungal infections of the nails, in dental medicine, in cosmetics, various medicines and, of course, in food preparations [29].

The chemical composition of the essential oils consists of a mixture of compounds, in different percentages, which together give the specific bioactive properties. Thyme and oregano essential oils used were analysed with Agilent 6890 N GC-MS highlighting the main constituent compounds: carvacrol 57.4%, ɑ-terpinene 32.4%, o-cymol 3.9%, for thyme essential oil [30] and thymol 64.4%, carvacrol 27.6%, limonene 3%, for oregano essential oil [31]. These compounds compositions are in agreement with those found in literature [32].

### 2.4. Preparation of Electrospun Collagen Nanofibres Loaded with Essential Oils

Collagen hydrolysate was used in high concentration due to the associative properties of collagen hydroysate molecules with effect on increased viscosity and improved electrospinnable properties. Thus, as compared to the viscosity of 1.5 cP of a collagen hydrolysate solution of 10% concentration (as it was obtained after enzymatic hydrolysis), by concentration up to 60%, the viscosity increased 1082 times. As compared to gelatin, the collagen hydrolysate represents a more versatile, cheaper and available material with higher tensioactive properties due to the amphiphilic character, which allows to obtain emulsions [33]. Concentrated collagen hydrolysate contains less water as compared to gelatin solution [34,35] and is easily miscible with different active substances, in our case, by mechanical stirring (400 rpm) for 50 min with 10% essential oils, added dropwise.

Nanofibres based on collagen hydrolysate (P1) and collagen hydrolysate loaded with 10% essential oils (thyme-P2 and oregano-P3) were obtained by the electrospinning technique using Fluidnatek equipment made by Bioinicia, Spain. The optimal electrospinning parameters were set as follows: the potential applied between electrodes of 20–22 kV, the flow rate of 0.7–0.9 mL h^−1^ and the collector-needle distance of 10–13 cm. The experiments were conducted at a temperature of 20 ± 2 °C and 40 ± 4% relative humidity. The obtained nanofibres were collected on a cotton textile for wound dressing simulation and kept in medical grade paper bags at room temperature. For the following structural, morphological and functional investigations, the nanofibre mats were released from the deposition support.

### 2.5. Investigation Methods

#### 2.5.1. Loaded Efficiency (LE) of Essential Oils

Essential oils’ loading efficiency was measured by ultraviolet–visible (UV–VIS) spectroscopy. Approximately 10–20 mg of each electrospun material were dissolved in methanol (99%). The supernatant was obtained by centrifugation at 6000 rpm for 10 min, filtered (0.45 μm polytetrafluoroethylene filter, Whatman) and analysed at 290 nm wavelength. The equation used to measure the loading efficiency (LE) was the following:

(1) $$\mathrm{LE}\;\left(\%\right)=\frac{EO\;measured\;amount}{EO\;theoretical\;amount}\times 100\%$$

The theoretical amount was calculated based on the essential oil added to the electrospinning solution (10 wt.% with respect to collagen hydrolysate solution).

The essential oil calibration curves (0–20 mg·mL^−1^) were made by dissolving thyme and oregano essential oils in methanol.

#### 2.5.2. Determination of Total Phenolic Content (TPC)

Determination of total phenolic content (TPC) is based on the electron transfer from phenolic compound to the complexed Mo (IV) from Folin-Ciocalteu’s reagent [35].

For this test, the samples were prepared as follows: 2 mg·mL^−1^ essential oil was obtained by dissolving each essential oil into 10 mL methanol and completed with distilled water to 100 mL; 6.4 mg of each nanofibre sample was dissolved into 3 mL methanol at room temperature; collagen hydrolysate sample (control) was analysed without any modification. For assay, 240 μL Folin-Ciocalteu’s reagent and 3.6 mL distilled water were added to each 50 μL extract sample prepared as above. The resulting solution was mixed and allowed to stand for 5 min in darkness at room temperature, then 0.68 mL solution of 7.5% (w/v) sodium carbonate (Na_2_CO_3_ × 10H_2_O) were added. The obtained solution was gently stirred and kept for 30 min in darkness at 40 °C. Finally, the solution absorbance was measured at 740 nm against blank. 

Total phenolic content (TPC) expressed as mg of gallic acid equivalent per g of dry weight (GAE/g dw) was determined using the gallic acid calibration curve (used as standard) in methanol (0–2000 mg·L^−1^, R^2^ = 0.999).

All tests were conducted with at least three replicas per sample and were expressed as mean ± standard deviation (SD).

#### 2.5.3. DPPH Radical-Scavenging Assay

**Figure 1:**
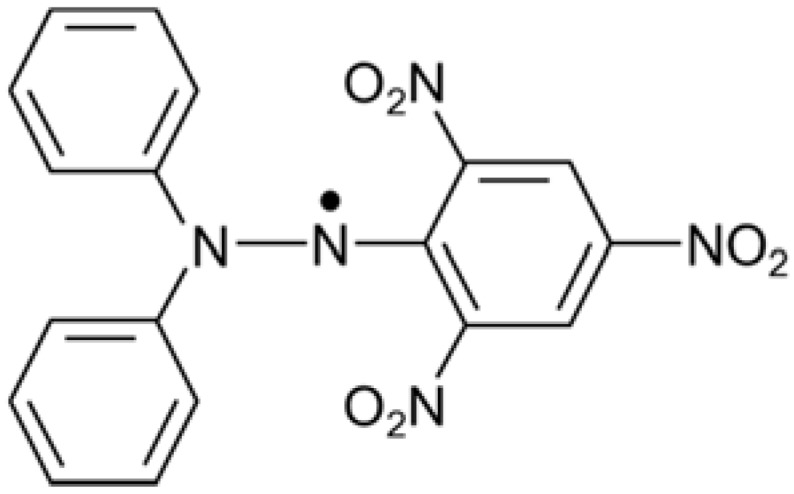
Chemical structure of 2,2-diphenyl-1-(2,4,6-trinitrophenyl)hydrazyl (DPPH) free radical.

The antioxidant activity of samples was determined using the 2,2-diphenyl-1-picrylhydrazyl (DPPH) method, based on neutralization of free radicals emitted by the DPPH (Figure 1) in methanol solution, resulting in a coloured solution. 

For assay, the stock sample solutions were prepared as follows: 2 mg·mL^−1^ of thyme essential oil and oregano essential oil, respectively, were obtained by dissolving 200 mg of each essential oil into 100 mL methanol; 0.2 g of concentrated collagen hydrolysate were diluted into 100 mL distilled water (corresponding to the concentration of collagen of 20 mg·mL^−1^); the nanospun samples, with (2 × 2) cm^2^ area were directly put into 2.5 mL DPPH solution. A total of 2.5 mL of DPPH in methanol solution (150 μmol·L^−1^) were mixed with 500 μL of stock sample solutions (above prepared) and after being left to stand 30 min in darkness at room temperature, then the absorbance was read at 517 nm, against blank, by using a UV/VIS spectrometer (Orion UV–VIS AQUAMATE 8000, Thermo Fisher Scientific). In addition, the affinity of nanospun samples to quench DPPH free radical was evaluated after 60 h in darkness at room temperature.

The quantity of DPPH radicals remaining in solution (RSA, %) was determined according to the Equation (2):

(2) $$\mathrm{RSA},\;\%=\left(\frac{A_{0}-A_{t}}{A_{0}}\right)\times 100\%$$

where *A*_0_ is the absorbance of DPPH solution without sample extract and *A_t_* is the absorbance of the sample extract.

The maximum effective concentration of collagen, thyme essential oil and oregano essential oil to inhibit 50% free DPPH radicals defined as IC50 was also estimated by testing different concentrations of essential oils in the range 1–10 μg·mL^−1^. The decreased values for IC50 signify a high antiradical efficiency.

All tests were conducted with at least three replicas per sample and were expressed as mean ± standard deviation (SD).

#### 2.5.4. ATR-FTIR Spectroscopy

The mid-infrared spectra of collagen nanofibres, essential oils and collagen nanofibres loaded with essential oils were analysed using an INTERSPEC 200-X spectrophotometer (Interspectrum, Estonia) with a device for attenuated reflectance (ATR). All spectra of the samples collected in triplicate were obtained in the wavelength range 4000 cm^−1^–700 cm^−1^, at a resolution of 2 cm^−1^. The ATR crystal was carefully cleaned with pure ethanol between measurements.

#### 2.5.5. Scanning Electron Microscopy (SEM) with Energy Dispersive X-Ray Analysis (EDX)

All microscopy images were examined using a FEI Inspect model S50 apparatus-Scanning Electron Microscope equipped with an EDX unit for elemental analysis. Secondary electrons (SE) images and Energy-dispersive X-ray spectra (EDX) were obtained. The SEM images were obtained at a 10 mm working distance, at 5 kV acceleration voltage and for magnifications from 1000× up to 10,000×. The nanofibres thickness represents the mean diameter of minimum 50 nanofibres measurements and the results were processed using ImageJ software. The average fibre diameters and their standard errors were calculated using Origin 2016 built-in Gaussian fitting curve software. The EDX spectra are obtained for image magnification of 10,000×. Prior to any investigations, all the samples were coated with a thin Au layer (~5 nm), in order to avoid charging effects. The Au layer that covers sample surfaces is obtained using a sputtering Cressington 108 auto sputter coater device, equipped with a Cressington mtm 20 thickness controller.

#### 2.5.6. Biocompatibility Assay

The cell line of mouse fibroblast (NCTC clone 929) was used (from the European Collection of Cell Culture—Sigma-Aldrich, USA) to determine the cell viability by the MTT test. This spectrophotometric method is a sensitive indicator of the cellular metabolic activity for detection of cell proliferation as it measures the reduction of a 3-(4,5-dimethylthiazol-2-yl)-2,5-diphenyltetrazolium bromide (MTT) into an insoluble formazan product by the mitochondria of viable cells. Fibroblast cultures (NCTC), were grown in Minimum Essential Medium (MEM) containing 10% fetal bovine serum (FBS) and Penicillin-Streptomycin-Neomycin (PSN). The NCTC cell line was inoculated at 4.0 × 10^4^ cells mL^−1^ density. The cells cultured in standard conditions, adhered to electrospun mats after 24 h of incubation, then the medium of culture was changed with a medium containing various concentrations of samples (100, 500, 750, 1000 μg·mL^−1^). The plates were incubated for 24 and 48 h at 37 °C, in 5% CO_2_ air atmosphere. The culture control was untreated cells cultivated in MEM and 10% FBS, and the positive control was H_2_O_2_ (2 μL·mL^−1^). After incubation with MTT solution (3 h, at 37 °C), the plates were placed on a shaker for 15 min and the absorbance was read at OD = 570 nm, using 96 wells microplate (Sunrise Tecan, Austria). All analyses were performed in triplicate.

Cell viability was determined by MTT colorimetric method. Thus, after removing the samples from the wells, the MTT solution (50 μg·mL^−1^) in MEM was added. After an incubation period of 3 h at 37 °C, in the atmosphere with 5% CO_2_, the MTT solution was removed and an equal volume of isopropyl alcohol was added. The absorbance was measured at 570 nm, using a Mithras LB 940 plate reader (Berthold Technologies). The results were reported as percentages of viability according to the control (cells incubated without samples) considered 100% viability.

For cell morphology analysis, the culture of mouse fibroblast cells (NCTC), fixed in methanol and Giemsa stained, was observed after 48 hours from the addition of the samples, and acquired with Zeiss AxioStar Plus microscope equipped with digital camera driven by AxioVision 4.6 software (Carl Zeiss, Germany).

#### 2.5.7. Determination of Antimicrobial Activity 

The antimicrobial assay was carried out on collagen based nanofibres samples (P1, P2, P3) suspended in dimethyl sulfoxide (DMSO) in concentration of 10 mg·mL^−1^ and compared with gentamycin/amphotericin (40 mg·mL^−1^/250 μg·mL^−1^), thyme and oregano essential oils (3 mg·mL^−1^) and undiluted DMSO.

The antimicrobial assay was performed using standard strains from the University of Bucharest, Microbiology Department collection, as follows: *Staphylococcus aureus* ATCC 25923, *Escherichia coli* ATCC 25922, *Pseudomonas aeruginosa* ATCC 27853 and *Candida albicans* ATCC 10231.

The qualitative screening of the antimicrobial properties was performed by an adapted spot diffusion method [36,37]. Bacterial and yeast suspensions of 1.5 × 10^8^ CFU·mL^−1^ (corresponding with 0.5 McFarland standard density) obtained from 24–48 h microbial cultures developed on Muller Hinton agar (MHA) and Sabouraud agar (SA) media, were used in the experiments. Petri dishes with MHA (for bacterial strains) and SA (for yeast) were seeded with microbial inoculums and an amount of 10 μL solution of each sample in concentrations mentioned above was spotted. The negative control was represented by dimethyl sulfoxide (DMSO). The plates were left at room temperature to ensure the equal diffusion of the compound in the medium and then incubated at 37 °C for 24–48 h. Sensitivity was evaluated by measuring the diameters of the inhibition zones that appeared around the spot and expressed as follows:

“−” absence of clear inhibition zone; “+/− −” very weak zone of inhibition; “+/−” weak zone of inhibition and “+” clear zone of inhibition.

The quantitative assay was performed in Sabouraud broth medium for yeasts and Muller Hinton broth for bacteria, using the binary serial microdilution technique on 96-well microtiter plates, according with Performance Standard for Antimicrobial Susceptibility Testing [38]. For establishing the minimum inhibitory concentration (MIC) values of the obtained solutions, a microdilution method performed in nutritive broth was utilized. The sterile broth was added in sterile 96 well plates and binary dilutions of each tested solution were performed in a final volume of 150 μL, followed by the addition of 15 μL microbial suspension adjusted to an optical density of 0.5 McFarland (1.5 × 10^8^ CFU·mL^−1^) in each well. The MIC values were established by visual analysis and spectrophotometric measurement (absorbance reading at 600 nm). Each experiment was performed in triplicate and repeated on at least three separate occasions.

For the quantitative assessment of the inert substratum adherence, in order to determine the minimal concentration for biofilm eradication values (MCBE), 96-multi well plastic plates containing binary dilutions of the tested compounds, in a final volume of 150 μL broth media, were inoculated with 15 μL microbial suspensions of 10^7^ CFU·mL^−1^ and incubated for 24 h at 37 °C. After incubation, the wells were discarded, washed three times by phosphate buffered saline (PBS) and the bacterial cells adhered to the plastic walls were stained by 1% violet crystal solution, for 15 min. The coloured adherent cells were thereafter fixed by cold methanol for 5 min and re-suspended by 33% acetic acid solution [39]. The absorbance at 490 nm of the blue suspension was measured using BIOTEK SYNERGY-HTX ELISA multi-mode reader, the obtained values being proportional with the number of the adhered microbial cells. The measurements were performed in triplicate.

#### 2.5.8. Statistical Analysis

Cell culture and bacterial studies were analysed by one-way analysis of variance (ANOVA) and Honestly Significant Difference (HSD) (95% significant level). In all cases, statistical significance was accepted when the probability (*p*) values were lower than 0.05.

## 3. Results

### 3.1. Characterization of Concentrated Collagen Hydrolysate

The physico-chemical properties of concentrated collagen hydrolysate are presented in Table 1.

**Figure 2:**
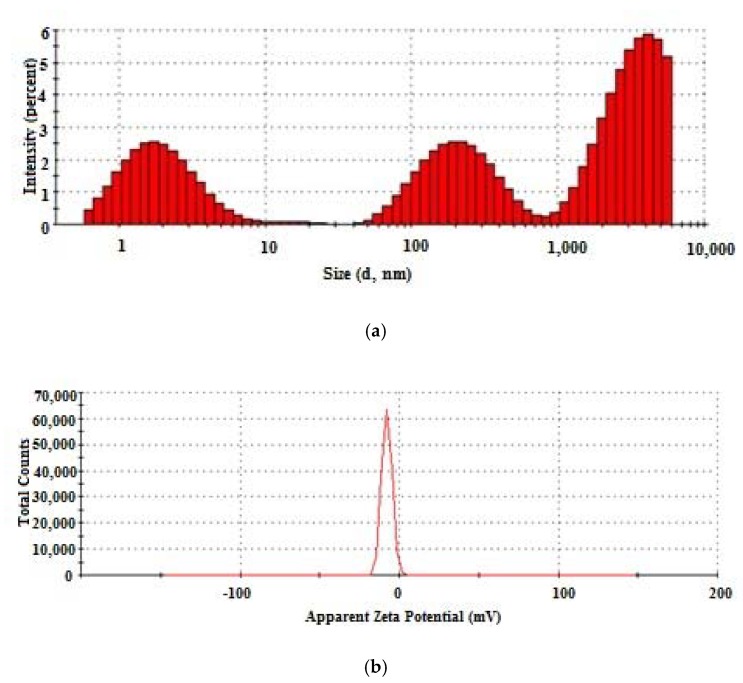
Histogram of particle size distribution (**a**) and zeta potential (**b**) in concentrated collagen hydrolysate.

The concentrated collagen hydrolysate is a polydispersion (Figure 2a) composed of three major particle populations of 3344 nm (46.7%), 236.2 nm (27.2%) and 2.4 nm (26.1%), with low stability and Zeta potential of −7.64 mV (Figure 2b).

### 3.2. Efficiency of Essential Oils Loading

It was found that the amounts of thyme essential oil and oregano essential oil loaded collagen were 29 ± 0.2 mg·mL^−1^ and 39 ± 0.4 mg·mL^−1^, respectively. These values are lower than the theoretical one (100 mg·mL^−1^), this means that the loading efficiencies were 29% and 39%, respectively. It is known that the boiling point of thyme essential oil (190 °C) is lower than that of oregano essential oil (239 °C) [40] and this can explain the difference of loading concentrations of these essential oils. A similar result (21–47% efficiency to encapsulation) was reported by Hosseini et al. [41] during nanoencapsulation of oregano essential oil into chitosan. The difference could be also attributed to some essential oil volatilization during the electrospinning process [42]. Collagen with smaller particle size (26.2% of 2.4 nm and 27.2% of 236.2 nm, as DLS analyses showed) would have a greater surface-to-volume ratio, thus may result in fast release of essential oil adsorbed into the collagen hydrolysate particle surface. The surfactant properties of collagen hydrolysate can generate an emulsion of essential oil in water with influence on their volatility. However, the encapsulation of essential oils into polymeric matrix was reported as an effective way for their long lasting storage [43]. 

### 3.3. Total Phenolic Content (TPC)

**Figure 3:**
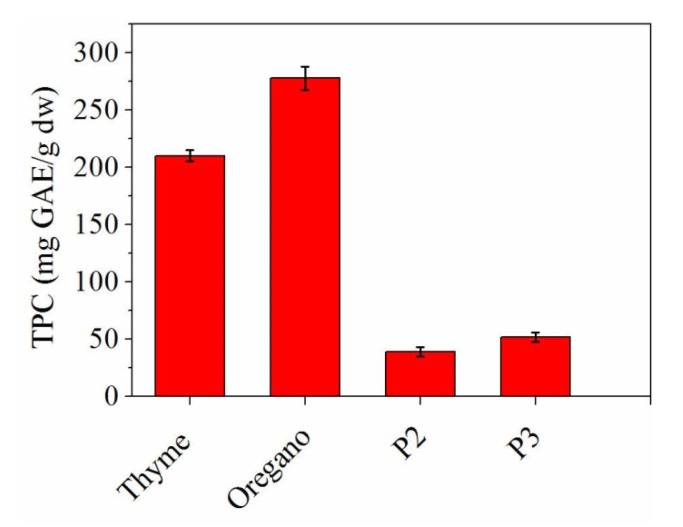
Total phenolic content for thyme essential oil, oregano essential oil and nanofibres based on collagen and essential oils.

Total phenolic content of essential oils and loaded essential oils into collagen hydrolysate samples is presented in Figure 3.

The concentrations of total phenol content in essential oils were found to be 277.74 ± 10 mg·GAE/g dw for oregano and 210.24 ± 5 mg·GAE/g dw for thyme. In Figure 3 it can be seen that the electrospun nanofibres contain phenolic compounds originating from thyme and oregano essential oils. The total phenolic content is higher in the electrospun collagen loaded with oregano essential oil (51.98 ± 4 mg·GAE/g dw) than that from the electrospun collagen loaded with thyme essential oil (39.12 ± 4 mg·GAE/g dw), in good agreement with the essential oils loading efficiency results. 

### 3.4. DPPH Radical Scavenging Activity Assessment

**Figure 4:**
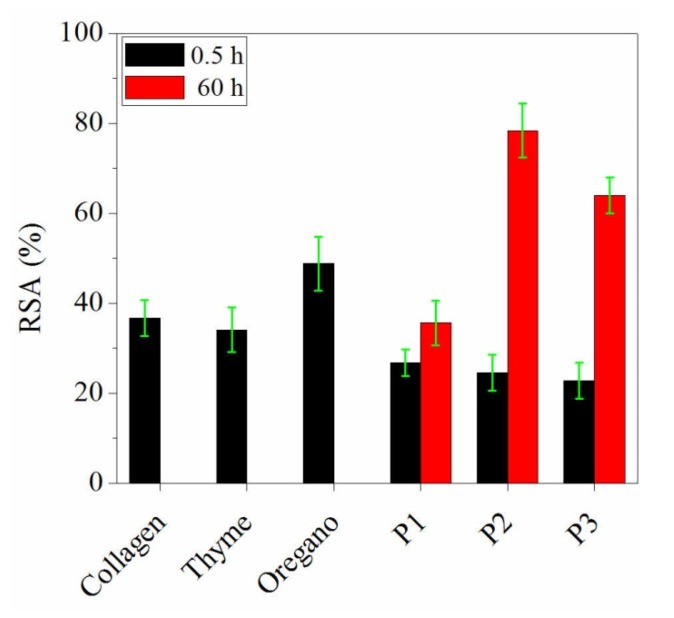
The DPPH radical-scavenging activity for collagen, thyme essential oil, oregano essential oil and nanospun samples (P1, P2 and P3).

The ability of tested component materials and prepared nanofibres to scavenge DPPH radicals is shown in Figure 4.

The antioxidant activity of collagen, thyme essential oil and oregano essential oil was in agreement with their TPC [35]. Collagen hydrolysate sample (20 mg·mL^−1^) showed a high ability to inhibit DPPH radicals (36.7 ± 4%) comparable with that of 2 mg·mL^−1^ thyme essential oil (34.1 ± 5%), explained by the high concentration of collagen in sample. 

The IC50 values for thyme and oregano essential oils were found to be 1.6 μg·mL^−1^ and 1.8 μg·mL^−1^, respectively.

No significant difference in RSA% of collagen nanofibres loaded with essential oils in comparison with nanospun collagen was recorded after 0.5 h. The results obtained could be attributed mainly to collagen. Nanospun collagen can act as a releasing agent of antioxidant compounds able to diminish the antioxidant activity of essential oils, probably due to the stabilization of phenolic compounds [44] by potential combination of these with free amino groups.

Instead, after 60 h, the nanospun collagen loaded with thyme essential oil showed the stronger antioxidant capacity (78.4 ± 6%) due mainly to carvacrol, followed by nanospun collagen loaded with oregano oil (64 ± 4%) than that of nanospun collagen (35.6 ± 5%). This behaviour could be assigned to the singular scavenging capacity of individual phenols [45] contained in essential oils. Nanospun essential oils loaded collagen can act as a high releasing agent of antioxidant ingredients both from collagen and encapsulated essential oils, to prolong the storage conditions.

### 3.5. Structural Analysis by ATR-FTIR

**Figure 5:**
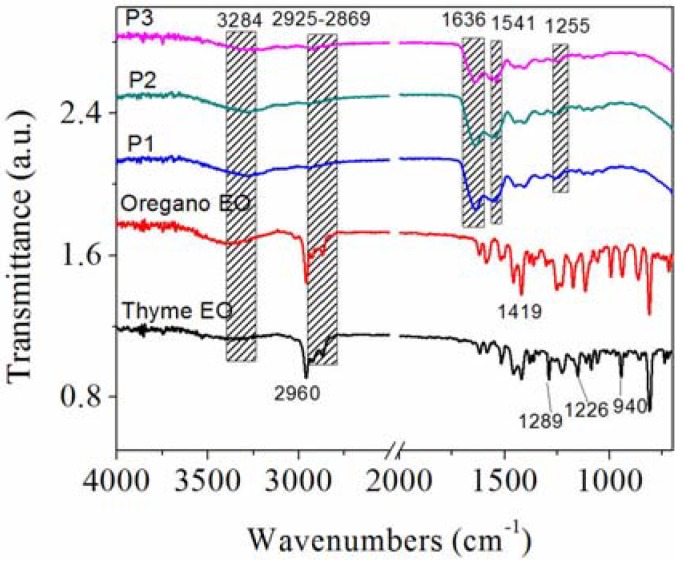
Normalized ATR-FTIR spectra for thyme essential oil, oregano essential oil, collagen nanofibres and collagen loaded with essential oils.

The FTIR analysis aimed at identification of thyme and oregano essential oils in electrospun samples and the possible interactions of these with collagen hydrolysate. ATR-FTIR spectra for thyme essential oil, oregano essential oil, collagen nanofibres and collagen loaded with thyme essential oil and oregano essential oil are presented in Figure 5.

The peaks that are common in the FT-IR spectra for thyme and oregano essential oils are ascribed at: 2960 cm^−1^ to antisymmetrical –CH_3_ stretching vibration, 1419 cm^−1^ to antisymmetrical –CH_3_ bending and 1226–1289 cm^−1^ to C–O–C stretching [46] and 940–945 cm^−1^ ɣ-terpinene [47].

Collagen nanofibres (P1) show amide A band at 3284 cm^−1^, associated with the stretching vibrations of N–H groups, amide I band around 1636 cm^−1^ (stretching vibrations of peptide C=O groups), amide II (around 1541 cm^−1^, N–H bending vibrations coupled to C–N stretching vibrations) and amide III (around 1255 cm^−1^, C–N stretching and N–H bending vibrations of amide linkages) [12]. 

The band at 2925 cm^−1^, characteristic of the asymmetric and symmetrical vibrations of the CH_2_ group present in essential oils was also found in collagen nanofibres loaded with thyme and oregano essential oils, respectively, but with low intensity. Absorption band displacement from 2869 cm^−1^ in oregano essential oil was evidenced in the P3 sample, as a result of the interaction between the hydrophobic groups present in the collagen and oregano essential oil.

By incorporation of thyme and oregano essential oils into collagen hydrolysate (P2 and P3 samples), both spectra show the typical pattern of protein molecules, suggesting that the prominent bands characteristic of essential oils were overlapped with the characteristic bands of collagen. 

### 3.6. SEM/EDX Analyses

**Figure 6:**
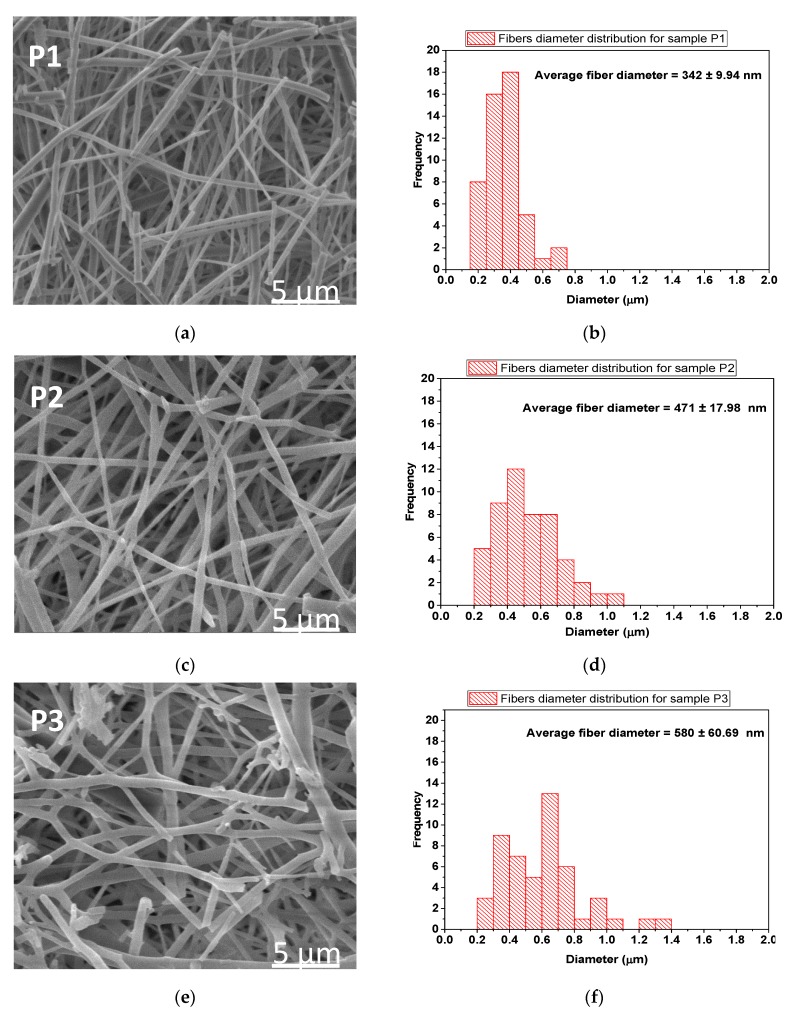
Scanning Electron Microscopy (SEM) images for P1 (**a**), P2 (**c**) and P3 (**e**) electrospun samples (magnification 10 kX) and fibre size distribution (**b**,**d**,**f**).

According to the SEM images (Figure 6), the average fibre diameters of electrospun collagen nanofibres (P1), electrospun collagen loaded with thyme essential oil (P2) and electrospun collagen loaded with oregano essential oil (P3) are: 342 ± 9.94 nm, 471 ± 17.98 nm and 580 ± 60.69 nm, respectively. The influence of essential oils loaded collagen is marked by the increased dimensions of electrospun samples in correlation with their total phenolic content results (Figure 3). Electrospun collagen loaded with oregano essential oil has the highest diameter (580 ± 60.69 nm), in agreement with the highest total phenolic content (51.98 ± 4 mg·GAE/g dw TPC), which means that the nanofibre thickness is influenced by the essential oil.

**Figure 7:**
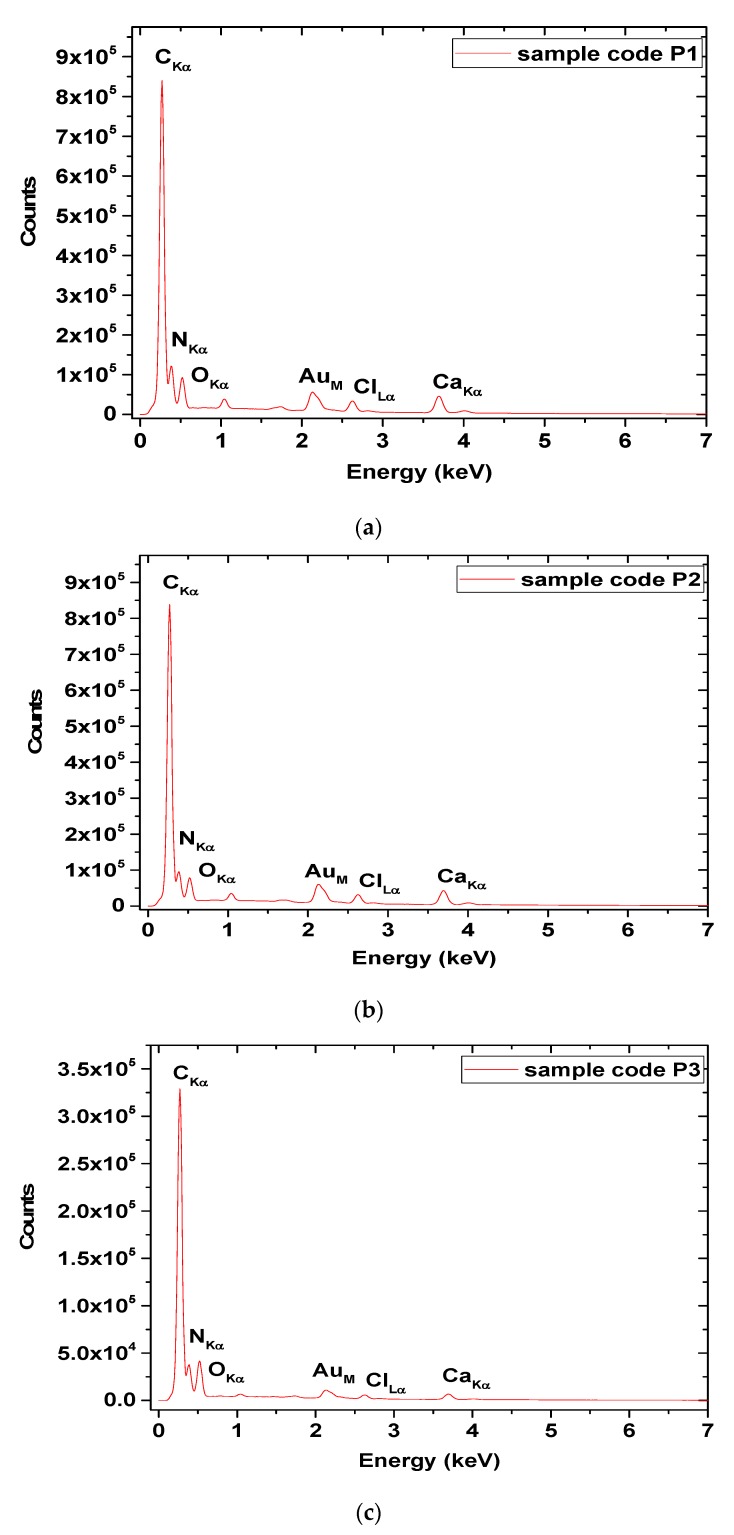
EDX analyses for the P1 (**a**), P2 (**b**) and P3 (**c**) electrospun samples.

EDX analysis for P1, P2 and P3 electrospun samples is presented in Figure 7, while the mass and atomic compositions are shown in Table 2.

From Table 2, more carbon is observed in electrospun collagen loaded with essential oils as compared to the collagen nanofibres and more carbon concentration in P3 as compared to P2, in agreement with LE and TPC results. Traces of chlorine and sodium are originated from collagen hydrolysate preparation. We highlight that the presence of Au is due to sample preparation prior to SEM/EDX measurement and not to the sample synthesis procedure, thus Au mass and atomic composition were excluded from the table.

### 3.7. In Vitro Evaluation of Collagen and Collagen Loaded with Essential Oils Nanofibres Biocompatibility

In vitro evaluation of the cytotoxicity effect of collagen and collagen loaded with essential oils nanofibres was performed on the stabilized cell line NCTC clone 929, both quantitatively (spectrophotometric—MTT) and qualitatively (optical microscopy methods).

**Figure 8:**
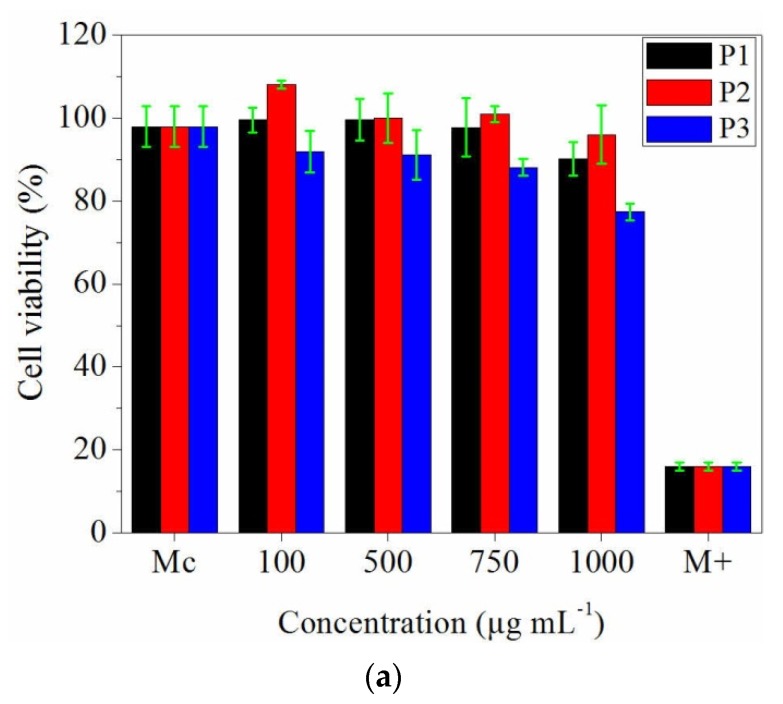

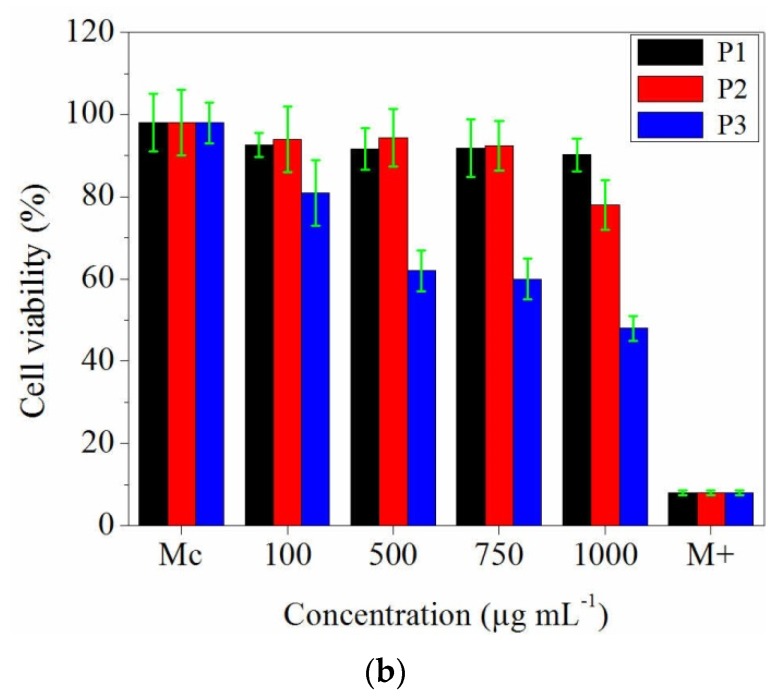
Cell viability of fibroblast cell line (NCTC clone L929) in the presence of prepared electrospun collagen and collagen loaded with essential oils after: (**a**) 24 h; (**b**) 48 h. P1 = collagen; P2 = collagen + thyme essential oil; P3 = collagen + oregano essential oil. Error bars represent the standard deviation. Mc = control, M+ = positive control.

The quantitative results of the 24 h cytotoxicity test have been represented in the Figure 8a. It can be observed that all analysed samples have no significant cytotoxic effect in the rage of 100–750 μg·mL^−1^ concentration, the viability of the tested cells being maintained at values between 99.58% and 90.23%. Moreover, in the presence of 100 µg·mL^−1^ of P2 sample, the cells proliferated more than the control (108.08%). The highest cytotoxic effect on the NCTC cell line was recorded for P3 sample at 1000 µg·mL^−1^ concentration, with 77.42% cell viability percent (Figure 8a).

After 48 h of contact, it was observed that the cell viability was maintained at higher percentages for P1 and P2 samples, in the concentration range 100–750 µg·mL^−1^. Instead, P2 sample showed a cytotoxic effect when its concentration was higher than 500 µg·mL^−1^, the cytotoxic effect being expressed by significantly decrease of the viable cells percentage, compared to the control (Figure 8b) (*p* < 0.01).

**Figure 9:**
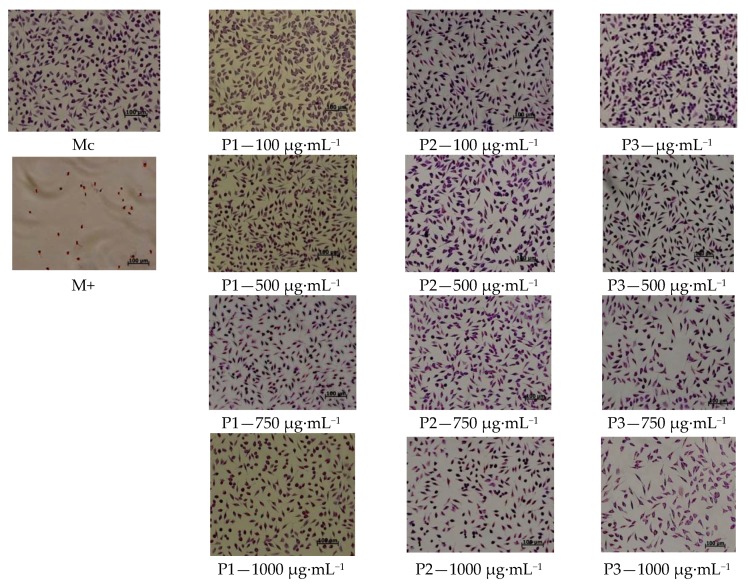
Cellular morphology after interaction with different concentrations for the P1, P2 and P3 samples (magnification 20×).

The mouse fibroblast cell line type NCTC morphology determined by Giemsa staining and microscopic analysis, allowed to evaluate the morphological and structural changes of the cells, after the contact with the tested samples (Figure 9).

In the Figure 9, it can be observed that the cells morphology was not modified when the cells were treated with P1 sample at 100–1000 µg·mL^−1^ concentrations and P2 sample (collagen loaded with thyme essential oil nanofibres) at 100–750 µg·mL^−1^ concentrations, comparing with the control (Mc). The cells present a uniform morphology, the cytoplasm is monochrome, without cell debris, demonstrating no cytotoxicity. For P3 sample (collagen loaded with oregano essential oil nanofibres), the same interaction was microscopically observed for 100 µg·mL^−1^ and 500 µg·mL^−1^ concentrations, but starting with 750 µg·mL^−1^ concentration, the cell density was lower and slight cell debris were observed, suggesting moderate cytotoxic effect (Figure 9).

### 3.8. Antimicrobial Activity

#### 3.8.1. The Qualitative Screening of the Antimicrobial Properties

Comparing with the antimicrobial activity of gentamicin and amphotericin, the qualitative test demonstrated that the thyme essential oil loaded collagen nanofibres kept the inhibitory effect toward all tested strains, improving the biological properties of the collagen nanofibres (*p* < 0.05). The very weak antimicrobial activity of the collagen nanofibres (considered as a negative control) is due to dimethyl sulfoxide (DMSO) used for the solubilization process (Table 3).

#### 3.8.2. The Quantitative Assay Results

Minimal inhibitory concentration (MIC) values (µg·mL^−1^) were established using the binary serial microdilution technique, starting with 1% of essential oil concentration calculated in 1 µg·mL^−1^ solution of collagen nanofibres and collagen nanofibres loaded with oregano essential oil and with thyme essential oil respectively, after solubilization in DMSO.

**Figure 10:**
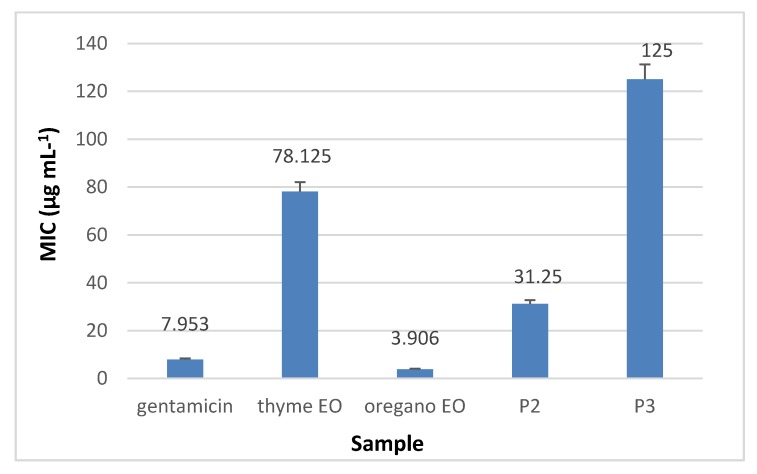
Minimum inhibitory concentration (MIC) of collagen nanofibres loaded with essential oils against *S. aureus* ATCC 25923, as compared to gentamicin and essential oils.

**Figure 11:**
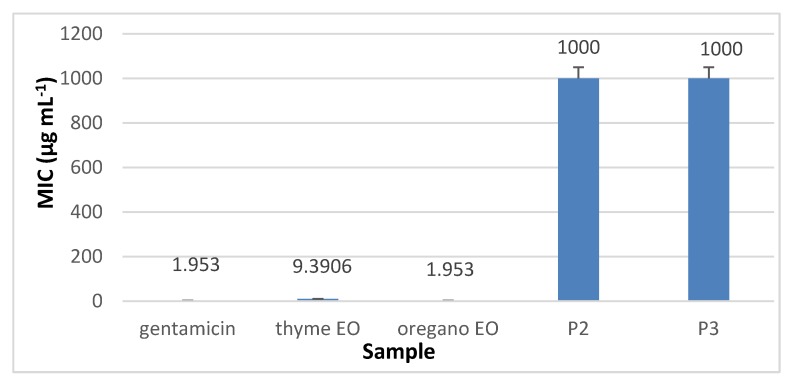
MIC of collagen nanofibres loaded with essential oils against *E. coli* ATCC 25922, as compared to gentamicin and essential oils.

**Figure 12:**
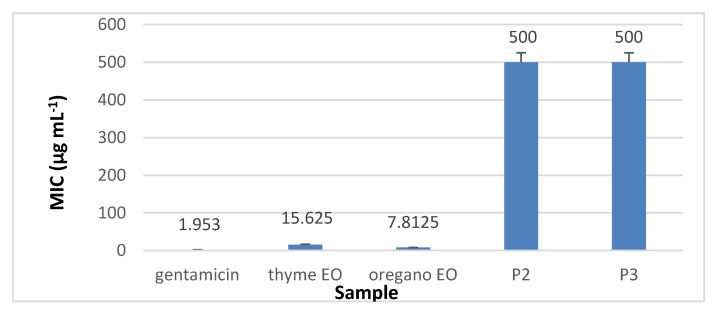
MIC of collagen nanofibres loaded with essential oils against *P. aeruginosa* ATCC 27853, as compared to gentamicin and essential oils.

**Figure 13:**
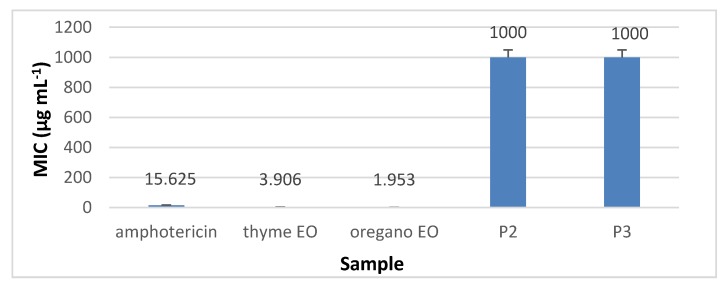
MIC of collagen nanofibres loaded with essential oils against *C. albicans* ATCC 10231, as compared to amphotericin and essential oils.

The results presented in Figure 10, Figure 11, Figure 12 and Figure 13 showed that the most sensitive strain to collagen nanofibres loaded with thyme essential oil and loaded with oregano essential oil respectively, is *S. aureus* ATCC 25923. MIC of collagen nanofibres loaded with thyme essential oil is 16 times higher as compared to thyme essential oil and 64 times higher as compared to gentamicin concentration (*p* < 0.05). Combinations of essential oils were tested against *S. aureus* ATCC 25923 with highest content of thyme essential oil, confirming the efficiency found in another study [48]. The MICs of collagen nanofibres against Gram-negative strains are higher as compared to the MICs against Gram-positive tested strain in agreement with essential oils behaviour. According to the literature reports, this behaviour is due to the lipopolysacharide membrane outer layer of Gram-negative strains which is not permeable for hydrophobic agents as compared to Gram-positive strains [49].

The most sensitive Gram-negative strain was *P. aeruginosa* ATCC 27853 at the same concentrations of collagen nanofibres loaded with thyme or oregano essential oils (Figure 12). The antibacterial activity of analysed oregano oil against *P. aeruginosa* ATCC 27853 was superior [48] or similar [50] to other results found in literature. MICs of 1 mg mL^−1^ of collagen nanofibres loaded with thyme or oregano essential oils showed to be efficient against *E. coli* ATCC 25922 and *C. albicans* ATCC 10231. 

MIC of collagen nanofibres for all tested strains was above 1 mg·mL^−1^ proving that the loaded essential oils supplemented them with antimicrobial properties, in agreement with other studies regarding the lack of antimicrobial properties of collagen materials [51]. Collagen nanofibres loaded with thyme essential oil showed to be the most efficient, taking into consideration the lower loaded concentration of thyme essential oil as compared to oregano oil.

#### 3.8.3. Minimal Concentration for Biofilm Eradication (MCBE)

**Figure 14:**
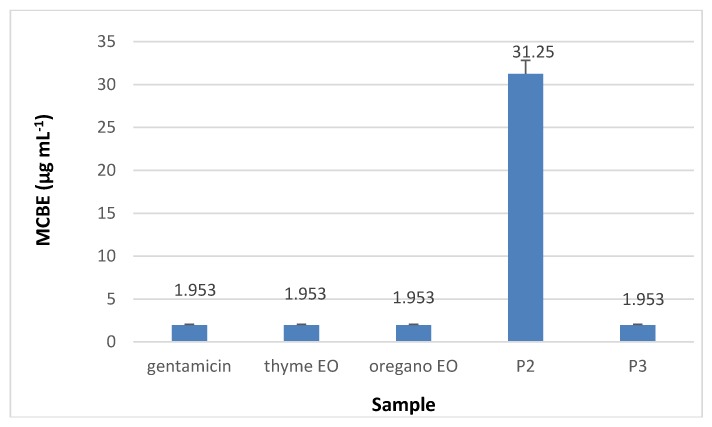
Minimal concentration for biofilm eradication (MCBE) of collagen nanofibres loaded with essential oils against *S. aureus* ATCC 25923, as compared to gentamicin and essential oils.

**Figure 15:**
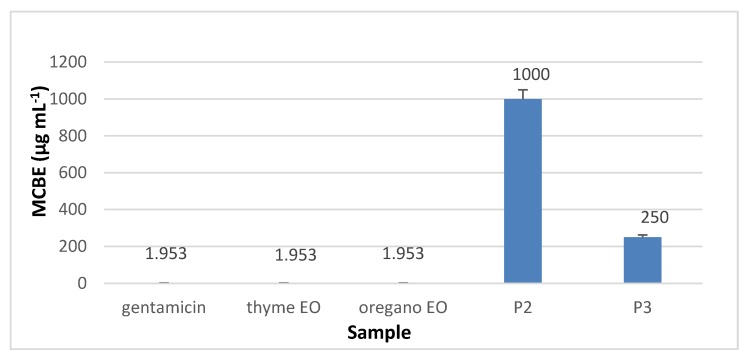
MCBE of collagen nanofibres loaded with essential oils against *E. coli* ATCC 25922, as compared to gentamicin and essential oils.

**Figure 16:**
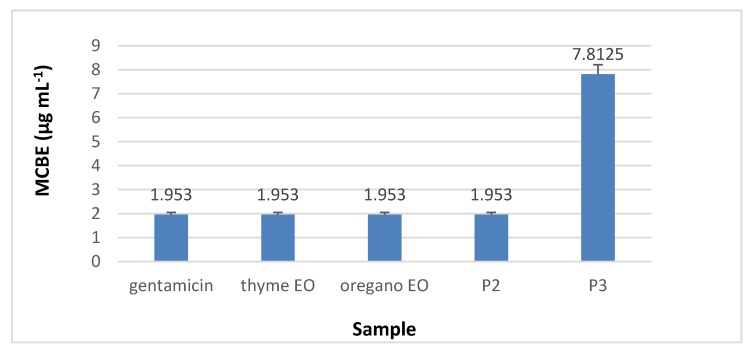
MCBE of collagen nanofibres loaded with essential oils against *P. aeruginosa* ATCC 27853, as compared to gentamicin and essential oils.

**Figure 17:**
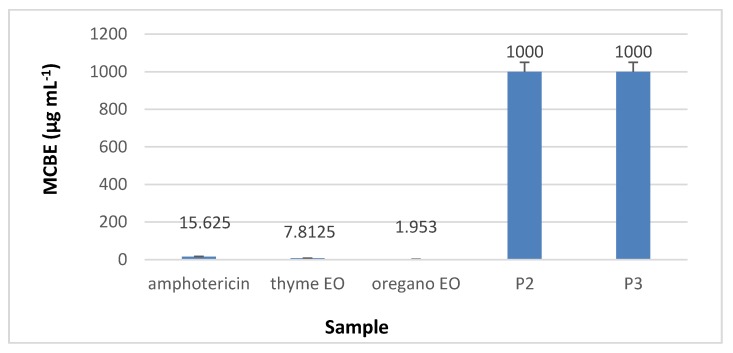
MCBE of collagen nanofibres loaded with essential oils against *C. albicans* ATCC 10231, as compared to amphotericin and essential oils.

Regarding the minimal concentration for biofilm eradication (MCBE) values, the analyses results showed that collagen nanofibres stimulated the microbial adherence to the inert substratum (MCBE > 1 mg·mL^−1^) while collagen nanofibres loaded with essential oils inhibited the biofilm development in the first 48 h, as can be seen in Figure 14, Figure 15, Figure 16 and Figure 17 (*p* < 0.01). Biofilm eradication effect was observed for *S. aureus* ATCC 25923 and *P. aeruginosa* ATCC 27853 at lower concentration than for *E. coli* ATCC 25922 and *C. albicans* ATCC 10231. Collagen nanofibres loaded with essential oils showed biofilm eradication properties, an essential factor for wound healing process. Recently, a new class of antibiotic resistance breakers (ARBs) was discovered based on the biofilm eradication ability which was found to be the most important stage in pathogen resistance reduction [52]. The need for new anti-biofilm strategies is recognized to be topical in the management of chronicle infections, the more so as 65% of bacterial infections are associated with the formation of biofim [53]. These results suggest that wound dressings based on collagen nanofibres loaded with essential oils can support and act in synergy with classical antibiotics against drug-resistant pathogens for healing of recalcitrant wounds. The positive influence of volatile essential oils on antibiotic activity enhancement was already reported [54].

## 4. Discussion

Commercially available antimicrobial dressings used to control the bacterial infections include antimicrobial agents such as antibiotics, quaternary ammonium salts, metal oxide nanoparticles and metal salt solutions [55]. Bioactive wound dressings have been developed to overcome toxic nature and reduced antimicrobial activity of classical wound dressings due to the antibiotics resistant pathogens.

In this context, nanofibre mats based on thyme or oregano essential oils loaded collagen hydrolysate with low cytotoxicity, antioxidant and antimicrobial properties were prepared by electrospinning. Electrospinning is a process that involves a high voltage supply, a syringe pump, a capillary tube with a needle and a manifold. For this, a polymer solution is pumped through a syringe; at the same time, a high voltage is applied to the spinneret to attract the drop to the collector. The main process parameters that influence the production of nanofibres are the feed rate, the applied voltage, the distance from the collector to the needle tip, the type of spinneret, the polymer and solvent properties [56]. Typical electrospinning parameters using collagen as a biopolymer were 0.3 mL·h^−1^ to 1 mL·h^−1^ for feed flow, voltage 10–25 kV and 7.5–13 cm for needle to collector spacing. However, the most common values were 0.7 mL·h^−1^, 19 kV and 13 cm for the feed flow, voltage and distance between the needle tip and the collector, respectively. The mixture of gelatin/PVA/keratin [57] was electrospun using 20 kV voltage, at 10 cm tip-to-collector distance and with a feed rate of 0.1 mL·h^−1^. In our research, we have exploited the molecule association property of collagen hydrolysate and the increase of polydispersion viscosity with concentration. We found that the viscosity of collagen hydrolysate increases 1082 times with increasing concentration 6.5 times. After many trials, we found that the collagen hydrolysate at the concentration of 60% in water, with and without 10% essential oils can be successfully electrospun by using typical parameters: the voltage of 10–25 kV, the distance of 7.5–13 cm from needle to collector spacing and the feed flow of 0.7 mL·h^−1^. Concentrated collagen hydrolysate in water represents an alternative to the use of more expensive native collagen or gelatin, usually solved in 1,1,1,3,3,3 hexafluoro-2-propanol (HFP), for tissue engineering applications. The use of acetone, ethylic alcohol as solvents or synthesis tensides for essential oils emulsifying in different polymer mixtures [25,33,34], glutaraldehyde for collagen nanofibres crosslinking [57,58] represents also more toxicological approaches as compared to proposed electrospinning process. The combination of concentrated collagen hydrolysate with essential oils for new wound dressing manufacture is an original and efficient approach for skin wound healing by cell proliferation stimulation and antimicrobial protection. Essential oils represent a natural alternative for synthesis antimicrobials with pathogen resistance potential for new non active wound dressings design.

The composite formulations based on natural materials like collagen hydrolysate and essential oils represent sustainable alternatives for more expensive native collagen, synthesis solvents, polymers, surfactants and antimicrobials with pathogen resistance and inflammatory potential [59].

Due to the associative properties of collagen hydrolysate, the viscosity of water polydispersion increased from 1.5 cP, the viscosity of 10% concentration polydispersion (resulted after the alkaline-enzymatic hydrolyses) to 1623 cP, the viscosity of 60% concentrated collagen hydrolysate, with improved electrospinnable properties. The essential oil emulsion formation was assisted by collagen hydrolysate tenside properties [28], which were shown to be superior to native collagen or aminoacids, with improved biocompatility as compared to synthesis surfactants [33]. 

The efficiency of essential oils loading was found to be 29% in the case of collagen nanofibres loaded with thyme essential oil and 39% in the case of collagen nanofibres loaded with oregano essential oil due to electrospinning conditions and essential oils characteristics. Similar encapsulation yields (21–29%) were reported for carvacrol included in starch or PCL matrices [60] or orange essential oil loaded gelatin (35–69%) [61], as a function of essential oil concentration. The total phenol content analysis carried out for thyme essential oil, oregano essential oil and nanofibres based on collagen and essential oils proved a higher value in the case of oregano essential oil incorporated into collagen, according to the total phenol content of tested essential oils. It was demonstrated that the high content of polyphenols will ensure the stabilization of collagen and protection against enzyme degradation [62].

Antioxidants assessment in terms of total phenolic content, and DPPH radical scavenging showed improved antioxidant activity of nanospun collagen loaded with essential oils due to the synergetic effect of collagen and loaded essential oils. Nanospun collagen loaded with essential oils can act as long lasting delivery support of antioxidant ingredients both from collagen and essential oils, to prolong the antimicrobial properties and storage conditions.

The ATR-FTIR analysis indicated that by incorporation of thyme and oregano essential oils into collagen hydrolysate (P2 and P3 samples) both spectra show the typical pattern of protein molecules, suggesting that the prominent bands characteristic of essential oils (2925 cm^−1^ with low intensity and absorption band displacement from 2869 cm^−1^ in oregano essential oil) were overlapped with the characteristic bands of collagen. The same behaviour was reported by other authors [22] in film-forming emulsion based on clove essential oil and melaleuca essential oil incorporated into chitosan.

SEM images showed that the bead-free and porous morphology of nanofibre mats are preserved but the collagen nanofibre diameter increased from 342 nm, to 471 nm for collagen nanofibres loaded with thyme essential oil and to 580 nm for collagen nanofibres loaded with oregano essential oil, in agreement with phenol content. The influence of essential oil on nanofibre diameter increase was found also for clove essential oil included in electrospun poly(ɛ-caprolactone)/gelatin nanofibres [63], fish oil encapsulated in poly(vinyl alcohol) nanofibres [64] and candeia essential oil included in polylactic acid nanofibres [65] and was attributed to electrical conductivity and viscosity changes. EDX analyses confirmed higher concentration of carbon in essential oil loaded nanofibres, with higher values for oregano oil loaded nanofibres.

Biocompatibility assay performed on NCTC clone 929 fibroblastic cells showed that the electrospun collagen nanofibres with thyme essential oil have slight and moderate cytotoxic effect only at 1000 µg·mL^−1^ concentration and the electrospun collagen nanofibres with oregano essential oil have slight and moderate cytotoxic effect at 500 μg·mL^−1^ concentration. As compared to other reported results related to the cytotoxicity concentration of 0.08 to 0.16 μL·mL^−1^ for thyme oil after 24 h [66], in our case the cytotoxicity limits of collagen nanofibres loaded with essential oils are very high.

Antimicrobial tests performed against *Staphylococcus aureus* ATCC 25923, *Escherichia coli* ATCC 25922, *Pseudomonas aeruginosa* ATCC 27853 and *Candida albicans* ATCC 10231, have shown antimicrobial efficiency, an important characteristic for wound healing process. Minimum inhibitory concentrations values of essential oils loaded collagen nanofibres presented strong antimicrobial activity against *Staphylococcus aureus* ATCC 25923 and *Pseudomonas aeruginosa* ATCC 27853 (MIC lower than 500 μg·mL^−1^) and moderate antimicrobial activity against *Escherichia coli* ATCC 25922 and *Candida albicans* ATCC 10231 (MIC ranging from 600 to 1500 μg·mL^−1^), if we take into consideration a proposed classification for antimicrobial activity of plant extract materials [67]. We have to mention that the same MIC value of 1 mg·mL^−1^, found for collagen nanofibres loaded with essential oils, was recorded for essential oil extracted from *Thymus ciliatus Desf*., tested for antifungal properties [68]. The minimum inhibitory concentrations of essential oils loaded collagen nanofibres against *Staphylococcus aureus* ATCC 25923 were lower (0.03125 mg·mL^−1^ and 0.125 mg·mL^−1^) than the value found for thyme oil against *Staphylococcus aureus* Rosenbach (0.25–4 mg·mL^−1^) reported by other authors [18].

Minimal concentration for biofilm eradication results confirmed the potential of essential oils loaded collagen nanofibres to be used for chronic wound treatments or supporting pathogenic resistant antibiotics. 

The association of collagen hydrolysate with essential oils cumulates the easily available, low molecular components for skin restructuring with natural antimicrobials in a completely biodegradable product. It is recognized that there are few studies regarding the inclusion of essential oils in electrospun fibres for wound dressings [63] and it is considered that the technology is recent, moreover the use of only natural materials without organic solvents or surfactants can be considered without correspondent.

Electrospun collagen nanofibres loaded with essential oils with bioactive properties can be used for obtaining bandages for wounds, clothing with specific properties, antimicrobial socks and gloves or in tissue engineering.

## 5. Conclusions

We have prepared and characterized a new type of bioactive nanofibres based on collagen hydrolysate loaded with essential oils (thyme or oregano). Collagen hydrolysate obtained by alkaline-enzymatic hydrolysis was concentrated and mixed in the right proportion with the essential oils to achieve the optimum parameters for obtaining antimicrobial nanofibres by electrospinning. ATR-FTIR spectra of functional groups and the UV-VIS spectroscopy based assessment of total phenolic content confirmed the essential oil loading of collagen nanofibres. Antioxidants evaluation in terms of DPPH radical scavenging showed that antioxidant properties of collagen was increased by essential oils which is a major advantage for antibacterial properties of new nanofibres. It was observed that the loaded concentration and the diameter of collagen nanofibres were influenced by the essential oil properties and electrospinning conditions. 

In vitro biocompatibility assay performed on NCTC clone 929 fibroblastic cells showed that the addition of essential oils to the obtained nanofibres does not induce cytotoxic effects under 500 μg·mL^−1^, cells showing a normal viability, morphology and development in the presence of the designed nanofibres.

Minimal concentration for biofilm eradication results confirmed the potential of collagen nanofibres loaded with essential oils to be used for chronic wound treatments or supporting pathogenic resistant antibiotics.

Electrospun collagen nanofibres loaded with thyme or oregano essential oils, with bioactive and biocompatible properties, were successfully obtained without organic solvents or surfactants and have potential for use in the medical, cosmetic or niche fields. Future studies will continue with kinetic of essential oil release from collagen nanofibres and the investigation of shelf life of products.

## Figures and Tables

**Table 1:** Physico-chemical characteristics of concentrated collagen hydrolysate.

Characteristics, U.M.	Value ± Standard Deviation
Dry matter, %	60.40 ± 0.42
Ash ^a^, %	6.24 ± 0.27
Total nitrogen ^a^, %	14.67 ± 0.66
Protein ^a^, %	82.43 ± 2.66
pH, pH units	8.54 ± 0.10
Aminic nitrogen ^b^, %	1.43 ± 0.06
Electrical conductivity, μs/cm	870 ± 0.1

^a^ values reported on dry matter; ^b^ value reported on the protein basis.

**Table 2:** Mass and atomic compositions for the P1, P2 and P3 electrospun samples.

Element	P1		P2		P3	
	Mass (%)	Atom (%)	Mass (%)	Atom (%)	Mass (%)	Atom (%)
Carbon	38.08	54.18	41.33	59.68	47.53	58.63
Oxygen	10.40	11.11	9.22	9.99	15.30	14.17
Nitrogen	19.38	23.66	15.75	19.50	21.85	23.11
Chlorine	4.24	2.04	4.34	2.12	2.09	0.87
Calcium	14.20	6.05	14.58	6.31	6.13	2.27
Sodium	1.78	1.32	1.63	1.23	0.73	0.47

**Table 3:** Expression of inhibition zone diameters for the tested microbial strains.

Expression of Inhibition Zone Diameters					
Samples	*S. aureus* ATCC 25923	*E. coli* ATCC 25922	*P. aeruginosa* ATCC 27853	Samples	*C. albicans* ATCC 10231
P1	+/− −	+/− −	+/− −	P1	+/− −
P2	+/−	+	+/−	P2	+
P3	+/−	+	+/−	P3	+
Gentamycin	+	+	+	Amphotericin	+

“−” absence of clear inhibition zone; “+/− −” very weak zone of inhibition; “+/−” weak zone of inhibition; “+” clear zone of inhibition.
